# Shifts in the clinical epidemiology of severe malaria after scaling up control strategies in Mali

**DOI:** 10.3389/fneur.2022.988960

**Published:** 2022-11-29

**Authors:** Drissa Coulibaly, Abdoulaye K. Kone, Bourama Kane, Bouréima Guindo, Bourama Tangara, Mody Sissoko, Fayçal Maiga, Karim Traore, Aichatou Diawara, Amidou Traore, Ali Thera, Mahamadou S. Sissoko, Ogobara K. Doumbo, Mark A. Travassos, Mahamadou A. Thera

**Affiliations:** ^1^Malaria Research and Training Center, Department of Epidemiology of Parasitic Diseases, Faculty of Medicine and Dentistry, University of Sciences, Techniques and Technologies of Bamako, Bamako, Mali; ^2^Service de Pédiatrie, Hôpital du Mali (HDM), Bamako, Mali; ^3^Center for Vaccine Development and Global Health, University of Maryland School of Medicine, Baltimore, MD, United States

**Keywords:** severe malaria, cerebral malaria, severe malarial anemia, seasonal malaria chemoprevention, Mali, epidemiology, malaria clinical phenotype

## Abstract

A decrease in malaria incidence following implementation of control strategies such as use of artemisinin-based combination therapies, insecticide-impregnated nets, intermittent preventive treatment during pregnancy and seasonal malaria chemoprevention (SMC) has been observed in many parts of Africa. We hypothesized that changes in malaria incidence is accompanied by a change in the predominant clinical phenotypes of severe malaria. To test our hypothesis, we used data from a severe malaria case-control study that lasted from 2014–2019 to describe clinical phenotypes of severe forms experienced by participants enrolled in Bandiagara, Bamako, and Sikasso, in Mali. We also analyzed data from hospital records of inpatient children at a national referral hospital in Bamako. Among 97 cases of severe malaria in the case-control study, there was a predominance of severe malarial anemia (49.1%). The frequency of cerebral malaria was 35.4, and 16.5% of cases had a mixed clinical phenotype (concurrent cerebral malaria and severe anemia). National referral hospital record data in 2013–15 showed 24.3% of cases had severe malarial anemia compared to 51.7% with cerebral malaria. In the years after SMC scale-up, severe malarial anemia cases increased to 30.1%, (*P* = 0.019), whereas cerebral malaria cases decreased to 45.5% (*P* = 0.025). In addition, the predominant age group for each severe malaria phenotype was the 0–1-year-olds. The decrease in malaria incidence noted with the implementation of control strategies may be associated with a change in the clinical expression patterns of severe malaria, including a potential shift in severe malaria burden to age groups not receiving seasonal malaria chemoprevention.

## Background

Malaria is one of the leading causes of morbidity and mortality in the world, with an estimated 3.3 billion people at risk of malaria (1). The incidence of malaria worldwide is estimated to be 219 million cases per year, with 92% of these cases occurring in sub-Saharan Africa. Between 2000 and 2019, revised estimated deaths due to malaria globally declined from 896 000 in 2000 to 558 000 cases in 2019. An increased to 627,000 deaths was reported in 2020 (1). Such deaths are principally due to two severe malaria subtypes: cerebral malaria—the deadliest clinical manifestation of malaria—and severe malarial anemia. The WHO African Region accounted for 96% of all deaths in 2019, mostly in children under 5 years of age. In Mali, West Africa, according to the local health information system, a total of 2,884,827 confirmed malaria cases were reported in 2020 with 871,625 severe cases, that resulted in 1,454 deaths (2).

In the past few decades, new tools, and strategies for malaria control such as artemisinin-based combination therapies (ACTs), long-lasting insecticide treated bed nets (LLINs), intermittent preventive treatment in pregnancy (IPTp), intermittent preventive treatment in infancy (IPTi) and seasonal malaria chemoprevention (SMC) in children have showed efficacy in malaria control (3, 4). Countries in which 80% or more among the target population benefited from these interventions reported a sharp decline in malaria burden (5).

In Mali, since 2007, public health efforts have provided children aged 0 to 5 years old with insecticide-treated mosquito nets and free treatment for malaria using artemisinin-based combination therapies (ACT). Seasonal malaria chemoprevention (SMC) was added starting in 2014. Scaling up of malaria control strategies (including the use of rapid diagnostic tests, ACTs, long-lasting insecticide-treated nets (LLIN), and SMC) has had an impact on the incidence of severe malaria and uncomplicated forms (6). In 2014, we initiated a case-control study of severe malaria with the aim to understand the role of human receptor-binding parasite proteins belonging to the *Plasmodium falciparum* erythrocyte membrane protein-1 (PfEMP1) family that are expressed on the surface of infected red blood cells. We investigated seroreactivity to specific variant PfEMP1 antigens to determine if seroreactivity was associated with an increased risk of cerebral malaria.

During the conduct of our study, we analyzed the distribution of severe malaria phenotypes and as reported here, noted a lower frequency of cerebral malaria cases in children than we had observed in previous studies. Such a finding, if confirmed, could have significant implications for malaria morbidity and mortality in Mali. We hypothesized that the scale-up implementation of effective strategies such as SMC might also affect the distribution of severe malaria clinical phenotypes. To test this, we analyzed the distribution of clinical phenotypes of severe malaria in hospital populations. We conducted a retrospective analysis of all cases of severe malaria hospitalized in the pediatric ward of the National Hospital of Mali (HDM) in the period 2013–2019. This time interval includes periods when SMC was implemented on a large scale (2016–2019) as well as the period prior to SMC (2013–2015).

## Methods

### Study design

We performed a multi-year severe malaria case-control study across multiple sites in Mali. Participants were enrolled and followed from October 2014 to December 2018. In addition, we collected data at the pediatric ward of the HDM, a national hospital located in the suburbs of Bamako. The HDM is one of three national referral hospitals in Mali with a specialized pediatric ward. Records from all children hospitalized for severe malaria in the period 2013 through 2019 were included if they fulfilled the study entry criteria.

### Study area

The study areas included the cities of Bandiagara, Bamako, Sikasso, and satellite villages located within an outer limit of 15 km around these cities (Supplementary Figure 1). The Bandiagara and Sikasso sites have hosted malaria field studies for more than two decades. In addition, they act as sentinel sites of the National Malaria Control Program for continuous surveillance of antimalarial drug efficacy. A description of the Bandiagara site is available elsewhere (7–9).

Sikasso is in the south of Mali, where *Plasmodium falciparum* is hyperendemic with seasonal peaks that occur in July-November and causes 95% of clinical malaria. Parasitemia prevalence rates range from 40 to 50% during the dry season (January–April) and 70 to 85% during the rainy season from May to December (10, 11). The main malaria vectors in the Sikasso region are *Anopheles gambiae* and *Anopheles funestus* with a sporozoite rate of 6.4% at the end of the rainy season and an entomological inoculation rate (EIR) of 0.032 infected bites per person per night (12). For Sikasso, there were three enrollment stations: i) the community health center of Bougoula-Hameau which is a peri-urban village of approximately 7,000 people located near the city of Sikasso, ii) the district Referral health center, and iii) the Hôpital Regional de Sikasso. These last two centers are in Sikasso town and are mandated to manage all pediatric health threats in the Sikasso region in their pediatric department.

The HDM is on the outskirts of Bamako city, where malaria transmission is hypoendemic. The pediatric department of the HDM manages severe pediatric health cases coming from various places, mostly from the Bamako suburbs.

Modalities of implementing malaria preventive measures in the study areas are described elsewhere (6). Briefly, in the period 2016–2018, nationwide mass distribution campaigns of Long-Lasting Impregnated nets (LLINs) were undertaken to target a ratio of one LLIN for two persons. Four rounds of SMC focused on children aged 3–59 months were completed in each year during the malaria transmission seasons. Insecticide Residual Spraying using Actellic 300 CS insecticide was done in 2017 and 2018.

### Participant recruitment and enrollment

#### Cases

Cases were recruited among children hospitalized or seeking care for severe malaria at the referral health care centers in the recruitment cities.

#### Controls

Each severe malaria case was matched by age class, residence, sex, and ethnicity to two uncomplicated malaria controls selected among children seeking care at the same health facilities, within a time window approximately 90 days from case enrollment.

#### Hospital cases

Clinical records from all children hospitalized in the pediatric ward of the HDM for severe malaria were included in the analysis.

#### Inclusion criteria

Participants meeting the inclusion criteria below were enrolled in the study after parents gave their informed consent.

Participants were enrolled if they were aged between 6 months and 10 years inclusive at the time of enrollment; had a confirmed *P. falciparum* malaria disease at enrollment; had residency in the vicinity of the study sites (Bandiagara, Sikasso or Bamako); written informed consent obtained from the parent/guardian; declared they were available to participate to the study follow-up schedule; and if those enrolled as cases had cerebral malaria with Blantyre coma score ≤ 2 and/or severe anemia with hemoglobin level ≤ 5 g/dl (13). In screening for severe malaria cases, we considered cases with any density of parasites to increase our sensitivity. In defining severe malaria, including severe malarial anemia, we did not limit ourselves to a specific threshold for peripheral parasitemia, knowing that severe malaria can be observed with a relatively low peripheral parasitemia. In rural areas such as our field sites, a patient may receive antimalarial treatment as self-medication that reduces parasitemia at the time of hospital admission.

The initial upper age limit for enrollment was 4 years of age but was eventually extended to 10 years.

For the HDM severe malaria case analysis, participants were aged 0 to 15 years old; permission from the HDM authorities was obtained before getting access to the patient's hospital records. Only completed records with a clinical diagnosis were included.

Participants were assessed for dehydration as recommended in World Health Organization guidelines for treating diarrhea (14). A child was considered dehydrated if the skin turgor was reduced, *i.e.*, if the skin of the abdomen gently pinched between the thumb and index finger kept the mark and took a few seconds to return to normal, associated or not with a dry mouth.

### Follow-up

Cases and controls were invited for a clinic visit on day 21 after their enrollment and in the following dry season for clinical examination and blood draws.

### Treatment

Cases were hospitalized and received treatment for severe malaria according to Malian National Malaria Control Program (MNMCP) and World Health Organization (15) Malaria Treatment guidelines. Artesunate was given intravenously or intramuscularly. If artesunate was not available, then intramuscular artemether or quinine was used. Oral treatment was given as soon as a child's clinical status was improved enough to make oral ingestion feasible. Uncomplicated malaria controls were treated as outpatients using combination artemether-lumefantrine medication.

### Laboratory assays

Venous blood was collected for molecular and immunological assays at enrollment, day 21, and the dry season visit. Thick smears, dry blood spots, hemoglobin level, glucose level were done on capillary blood collected by finger prick. At enrollment, lumbar puncture was performed on cases to exclude a diagnosis of bacterial meningitis.

### Malaria diagnosis, hemoglobin, and blood glucose

Giemsa-stained thick smears were obtained for malaria diagnosis at enrollment, day 21. and the dry season visit. Parasite density was quantified each time malaria smears were obtained. MRTC CAP-certified clinical laboratory standard operating procedures were followed to assure uniform and high-quality malaria smears. Thick smears were read by counting the number of parasites seen per 300 white blood cells. Parasite density was calculated based on an expected 7,500 white blood cells/mm^3^. Hemoglobin and blood glucose were determined for early diagnosis of severe anemia and low blood sugar by using hemoglobin and glucose analyzers (HemoCue^®^ Inc, (301 System), Cypress, CA), respectively.

### Sample size

The initial sample size was computed to allow enrollment of 35 cases and 70 controls per year for 4 years, to reach a total sample size of 420. Assumptions were based on a normal distribution of the proportion of expressed non-CD36-binding Plasmodium falciparum erythrocyte membrane protein-1s (PfEMP1s), with a 0.80 power to detect a difference of 2.5% in non-CD36-binding PfEMP1 expression. However, there were much fewer severe malaria cases than expected and as a result, we extended enrollment to additional sites, recruiting all cases of severe malaria that we encountered and that conformed to the study inclusion and exclusion criteria. In this manner, we achieved the reported sample size. While this sample size did not meet our original goal, it still allowed us to observe an increase in the frequency of severe malaria anemia that we deem useful to be shared with the scientific community.

### Ethical clearance

The study protocol was approved by the Ethics Committee of the Faculty of Medicine and Odonto-Stomatology/Faculty of Pharmacy, University of Sciences, Techniques and Technologies of Bamako, Mali; letter of approval #2014//97/CE/FMPOS. The study protocol also obtained approval from the University of Maryland School of Medicine Institutional Review Board. Permission to work in the communities was obtained from local officials and traditional authorities of the respective study settings (16). Annual continuing reports on the status of study implementation were submitted to both review boards and annual approvals to continue the study were granted through the end of the study in 2019. Individual informed consent was obtained from the parent/guardian. Data were anonymized to guarantee the confidentiality of participants' identities. All participants seen by the research medical team received free medical care, including treatments for concomitant diseases.

### Statistical methods

Data were collected onto individual Case Report Forms. DataFax version 14.1.2, a hybrid fax and electronic data capture system, was used. DataFax is a data management system designed to manage paper data forms, which are e-mailed (or faxed) from remote sites to a central computer running the DataFax system using a server. Data were then exported as Microsoft Excel version 2013; and analyzed using SPSS software, version 13 (SPSS, College Station, TX). Descriptive statistics were used to summarize baseline values and demographic characteristics (age, gender, ethnicity, and residency). Pearson chi-square tests or exact probability statistics were used to compare categorical variables.

## Results

The frequency of severe malaria cases for the case-control study was much lower than we had expected, and we ultimately enrolled all cases that fulfilled the inclusion criteria. Over the four years of the study, we enrolled 97 cases and 61 controls in total (Supplementary Figure 2). In the hospital dataset, we included a total of 1,338 records diagnosed with severe malaria: 633 records before SMC and 705 records after SMC was introduced at scale.

**Figure 1:**
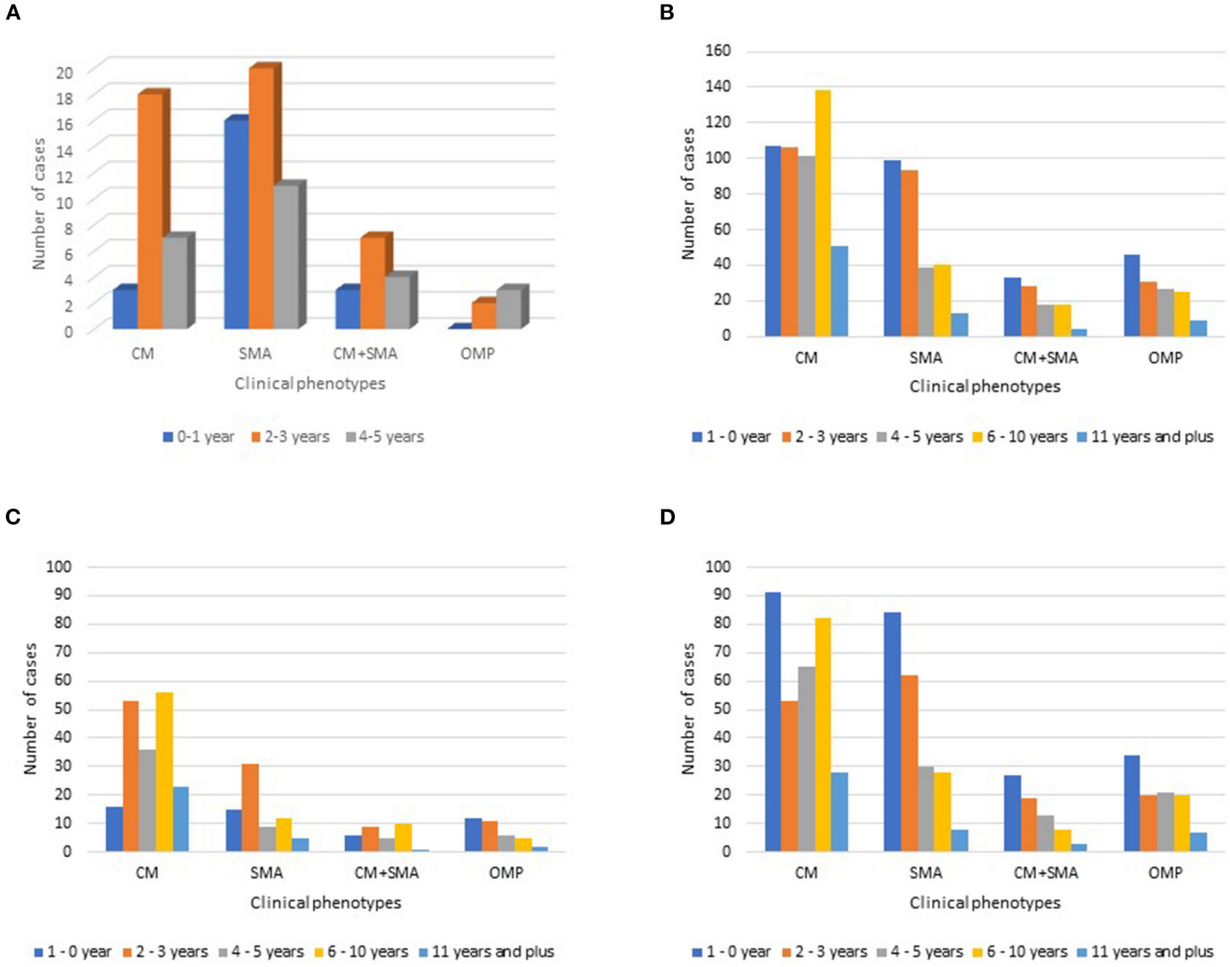
Severe malaria clinical phenotype distribution by age group for **(A)** the case-control study and for **(B)** the National Hospital of Mali record survey. Also included is the severe malaria clinical phenotype distribution by age group for the National Hospital of Mali record survey from 2013 to 2015 **(C)**, prior to seasonal malaria chemoprevention introduction, and from 2016 to 2019 **(D)**, after seasonal malaria chemoprevention was initiated. CM, cerebral malaria; SMA, severe malarial anemia; CM+SMA, concurrent cerebral malaria and severe malarial anemia; Other mixed phenotypes (OMP): any combination of signs and symptoms in a case confirmed to be severe malaria that is not CM+SMA.

In the case-control dataset, participants were roughly evenly divided by gender. Overall, the median age was between 3.16 to 3.41 years old (Table 1A). The highest number of cases fell within the 2–3-year-old age range (Figure 1A). In the hospital dataset, the median age increased after SMC, from 3.0 years old to 4.0 years old (*P* = 0.031; Table 1B).

**Table 1A:** Baseline characteristics of severe malaria case-control study participants.

	**Cases**	**Controls**	***P*-value**
	**(*N* = 97)**	**(*N* = 61)**	
**Median age in years**	3.16	3.41	0.18
	**Count (%)**	**Count (%)**	
**Residency**			
Bandiagara	37 (38.1)	46 (75.4)	< 0.05
Bamako	51 (52.6)	12 (19.7)	
Sikasso	8 (8.2)	3 (4.9)	
**Ethnicity**			
Bambara	26 (26.8)	8 (13.1)	0.01
Dogon	39 (40.2)	47 (77.0)	
Fulani	6 (6.2)	2 (3.3)	
Others	26 (26.8)	4 (6.6)	

**Table 1B:** Baseline characteristics of National Hospital of Mali severe malaria patients.

	**Cases before SMC**	**Cases after SMC**	***p*-value**
	**[2013–2015]**	**[2016–2019]**	
	**(*N* = 633)**	**(*N* = 705)**	
**Median age in years**	4.00	3.00	0.031
	**Count (%)**	**Count (%)**	
**Gender (male)**	333 (52.6)	418 (59.3)	0.014
**Ethnicity**			0.816
Bambara	348 (55.0)	382 (54.2)	
Dogon	44 (6.9)	54 (7.6)	
Fulani	89 (14.1)	90 (12.8)	
Others	152 (24.0)	179 (25.4)	

In the case-control dataset, most of the enrolled cases included a severe malarial anemia (SMA) component. Almost half of the children were diagnosed with severe malaria anemia alone. Cerebral malaria (CM) alone was diagnosed in approximately 30% of the cases. We also found a proportion of cases with both CM and SMA concurrently (14.4%; Table 2A). The case fatality rate of severe malaria was 10.3% (10/97). *P. falciparum* was the sole parasite species identified by microscopy (Supplementary Table 2).

**Table 2A:** Distribution of severe malaria clinical phenotypes in the case-control study.

	**Cases (*****N*** = **97)**	
	***n***	**%**
**Clinical phenotype**		
CM only	29	29.9
SMA only	48	49.0
SMA+CM	14	14.4
Others mixed phenotypes (OMP)	6	6.2

In the hospital dataset prior to SMC, one third of the children had severe malaria anemia. Most cases (51.7%) were classified as having cerebral malaria alone (Table 2B). The proportion of cases classified as cerebral malaria alone decreased in the SMC era, from 51.7% before SMC to 45.5% after SMC (*P* = 0.025). In contrast, the proportion of children classified with severe malaria anemia alone increased from 24.3 to 30.1% in the SMC era (*P* = 0.019). Approximately 10 percent of children had both CM and SMA concurrently, a statistic that remained unchanged in the SMC era (*P* = 0.764).

**Table 2B:** Distribution of severe malaria clinical phenotypes in the National Hospital of Mali record survey.

	**Pre SMC**		**Post SMC**		***p***
	**[2013–2015]**		**[2016–2019]**		
	**(*****N*** = **633)**		**(*****N*** = **705)**		
	***n***	**%**	***n***	**%**	
CM	327	51.7	321	45.5	0.025
SMA	154	24.3	212	30.1	0.019
SMA+CM	66	10.4	70	9.9	0.764
OMP	86	13.6	102	14.5	0.643

Other mixed phenotypes (OMP): any combination of signs and symptoms in a case confirmed to be severe malaria that is not CM+SMA. This includes respiratory distress+hypoglycemia; hypoglycemia+hyperparasitemia; and respiratory distress+hypoglycemia+dehydration.

The italic values indicate *p*-values.

**Figure 2:**
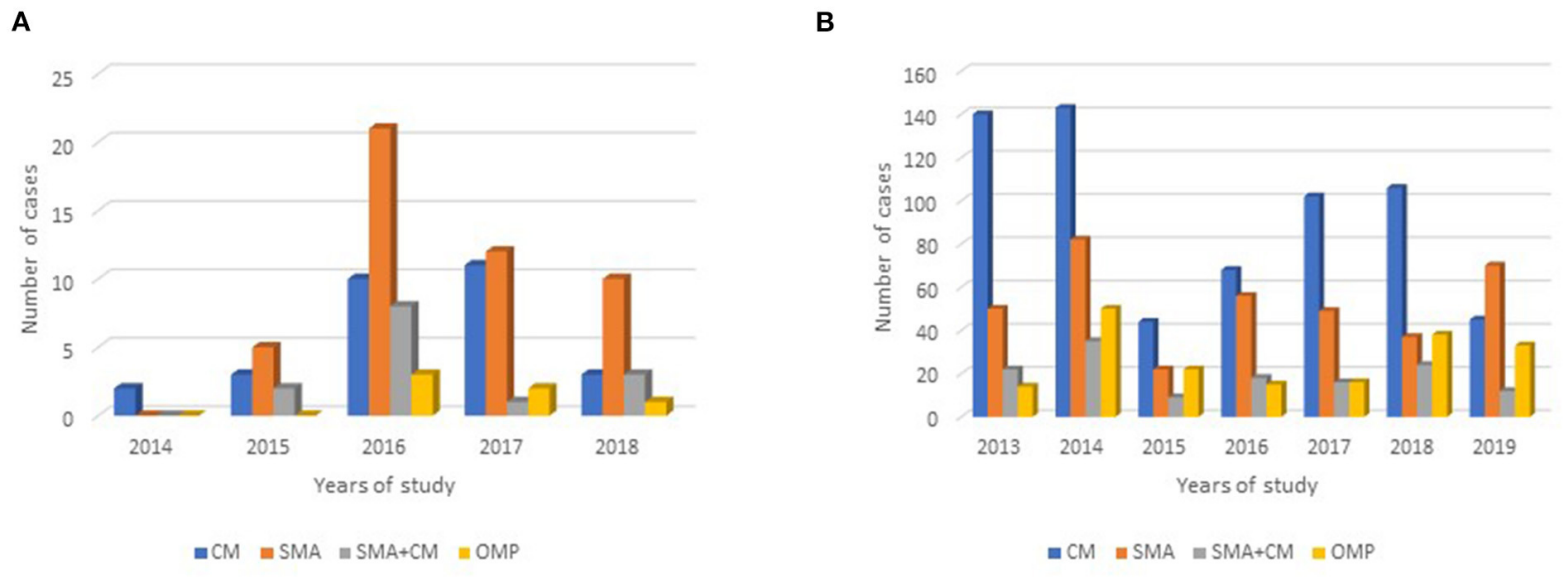
Distribution of severe malaria clinical phenotype by study year for the case for **(A)** the case-control study and **(B)** the National Hospital of Mali record survey. CM, cerebral malaria; SMA, severe malarial anemia; CM+SMA, concurrent cerebral malaria and severe malarial anemia; Other mixed phenotypes (OMP): any combination of signs and symptoms in a case confirmed to be severe malaria that is not CM+SMA. The italic values indicate *p*-values.

For the case-control study, except for the first year of the study—when enrollment was only two cases—severe malarial anemia alone was consistently the most severe malaria clinical phenotype encountered (Figure 2A, Supplementary Table 1). Most children with severe malaria anemia in the case-control study were three years old or younger (Figure 1A).

At HDM, children with CM or SMA alone included a broad range of ages. Most children with severe malarial anemia were under three years of age (Figure 1B). In comparison, children with cerebral malaria tended to be older, with the largest category of such children in the 6–10-year-old group (Figure 1B). In each year of the study from 2013 to 2018, the most common severe malaria diagnosis was cerebral malaria alone (Figure 2B, Supplementary Table 3). In contrast, in 2019, the most common severe malaria diagnosis was severe malarial anemia alone.

Prior to SMC, the predominant age group for each severe malaria phenotype was older than 1 year of age (Figure 1C). In the era following SMC introduction, the age group with the highest number of cases was the 0–1-year-old age group for each severe malaria phenotype (Figure 1D).

## Discussion

We analyzed the clinical phenotype of severe malaria cases in a case-control study undertaken in Mali in 2014 through 2019. We focused on cerebral malaria and severe malarial anemia, the two most frequently observed clinical phenotypes. We observed a greater frequency of severe malarial anemia and a lower-than-expected frequency of cerebral malaria cases. SMA represented half of all cases of severe malaria disease, while one-third of cases had a CM clinical phenotype. Severe malaria clinical cases were, overall, less frequent than expected among all hospitalized patients at health care centers where study cases were enrolled. In addition, we noted potential age shifts in severe malaria following the introduction of seasonal malaria chemoprevention.

We applied the World Health Organization 2014 definition (17) for CM and SMA in the case-control study. CM was diagnosed in children with Blantyre Coma Score (BCS) <3, in the presence of peripheral asexual parasitemia and no signs or symptoms of other clinical conditions. We excluded children presenting only with prostration, defined as impaired consciousness that did not reach the stage of deep coma or convulsions. This restricted case definition for CM substantially reduced our capacity to enroll a higher number of severe malaria cases. Some children may have presented prior to developing cerebral malaria, particularly those with clinical symptoms such as fever, impaired consciousness, or convulsions. These clinical signs or symptoms are likely to trigger the attention of parents and to lead to active care-seeking behavior. This likely prevented the child's illness from reaching a stage of deeper coma. This was supported by the high frequency of cases classified as CM in the HDM hospital dataset. This dataset reflects the routine practice of applying a more inclusive definition for CM than what we used in the case-control study, considering a case of CM as the presence of asexual parasitemia plus impaired consciousness, convulsions related or not to fever, or a coma with a Blantyre Coma Score <3.

For SMA, we applied the definition of a hemoglobin threshold rate of 5 g/dL with peripheral parasites and an absence of other causes that could explain the child's signs and symptoms. Cases of SMA tend to follow an insidious development with clinical signs and symptoms that slowly manifest and are harder to detect. Given the fact that anemia evolves with less visible clinical symptoms than coma, it is likely that parents seek care earlier in cases of cerebral malaria than severe malarial anemia.

To verify if the higher frequency of anemia among severe malaria cases was an artifact due to the design of our case-control study or whether it reflected a true shift in the clinical phenotype of severe malaria disease, we investigated the 2013–2019 hospital records in a pediatric ward that serve as referral center for a large portion of the suburbs of Bamako capital of Mali. This interval encompasses the period prior to SMC implementation. Overall, we observed a frequency of 51.7% of CM cases in the pre-SMC period and an intriguing decrease in the post-SMC period. We also observed a significant increase in the SMA case counts post-SMC (30.1%) compared to the pre-SMC (24.3%) period, but the percentage was still lower than what we observed in the case-control dataset. Despite the difference in case definitions, the findings in the hospital dataset mirrored what we observed in the case-control dataset, with a tendency for more SMA cases after SMC implementation at scale in an area of seasonal malaria transmission. This should lead us to investigate more carefully how clinical phenotypes of severe malaria are affected by effective control strategies.

Our data suggests a change in the relative frequency of the clinical phenotypes of severe malaria and a potential age shift in severe malaria vulnerability. This change coincides with the scale-up of implementation of efficacious control measures in Mali such as SMC that targets children 6–59 months old and the systematic use of Rapid Diagnostic Tests (RDTs), prompt treatment using efficacious ACTs, and vector control activities. Our hospital record survey noted that the predominant age group for each severe malaria phenotype following SMC introduction was 0–1-year-old. This suggests potential shifts in severe malaria burden to age groups not receiving seasonal malaria chemoprevention.

In African countries where SMC and other control strategies including ACTs, LLINs, IPTp and Intermittent Infant Preventive Therapy (IPTi) have been implemented on a large scale, a sharp reduction in the incidence of uncomplicated malaria as well as that of severe malaria has been reported (5). Beginning in 2016, the Mali NMCP implemented an active control strategy with the aim of achieving high coverage of the target population. This translated into sustained implementation of SMC countrywide as well as other malaria control approaches (18).

Recent studies have shown changes in susceptibility to malaria following the scaling up of control measures in Mali (6). These changes could also be associated with a shift in the clinical expression of malaria, especially for severe clinical phenotypes.

High malaria transmission has been associated with a concentration of most clinical malaria cases in younger children and with SMA as the most frequent clinical phenotype among cases of severe malaria (17). In areas with lower malaria transmission intensity such as Sahelian countries with seasonal malaria, most severe malaria cases present as cerebral malaria (17). Several malaria epidemiology studies have reported across Africa a higher frequency of SMA in countries with intense perennial malaria transmission (17) and more CM in countries with lower seasonal malaria transmission. We expect a lowering of malaria transmission with the implementation and scale-up of effective malaria control strategies. Moreover, in a case-control study of severe malaria conducted in Bandiagara in 1999–2001, several years before scaling up of malaria control interventions, we noted a frequency of 20.8% (*n* = 253) of SMA and 49.3% of CM (7). Our present findings in Bandiagara suggest that SMA has become more frequent there than CM. If confirmed by observational studies in other regions, this would constitute a significant shift in the epidemiology of severe malaria that trends in a direction opposite that expected with a decreased malaria transmission. One additional consideration is that anemia may be due to various etiologies including but not limited to infections, hematology disorders, and malnutrition. An increased frequency of any of these conditions could contribute to an increase in severe malarial anemia.

A reduction in cerebral malaria frequency could contribute to reductions in malaria morbidity and mortality. Cerebral malaria is the deadliest clinical expression of severe malaria in children, with close to 20% of cases leading to death (19). Early treatment could halve the case fatality rate; however cerebral malaria occurs typically in situations with delayed access to treatment. Recent studies in Malawi have shown that death due to cerebral malaria was associated with brain swelling and herniation into the spinal canal (20). In cases where children survived, brain damage often led to short- and long-term sequelae that included convulsions, epileptic seizures, and more subtle alterations of brain functions with various consequences (21). More detailed clinical evaluation of cerebral malaria in future studies has the potential to provide further insights into the changing nature of cerebral malaria in Mali. This study was conducted in remote sites with limited diagnosis capacity. Neither computed tomography nor magnetic resonance imaging was available, and there was no capacity to measure intracranial pressure with the lumbar puncture evaluation or measure other standard lumbar puncture indices outside of a bacteriologic evaluation.

The assessment of dehydration was based only on clinical judgment, mainly by assessing skin turgor, as serum electrolyte and lactate levels could not be assessed due to limited equipment and supplies. Neither the estimation of the proportion of weight change nor the measurement of the water volumes nor the electrolyte balance was performed to support the diagnosis of dehydration, as has been done in other severe malaria studies in Kenya and Gabon (22, 23). Abnormal skin turgor showed a high likelihood ratio of [LR 2.5 95%CI 1.5–4.2] to be predictive of dehydration in children (24).

This study was also limited by the low number of cases of severe malaria encountered in the case-control study period. This led to a study expansion from Bandiagara alone in 2014 to Bamako and Sikasso in the south of Mali. As the case-control study recruitment was contemporaneous with increased investment in malaria control country-wide, we observed an overall lower number of severe malaria cases. Another limit is the lack of comprehensiveness in the hospital records and the rather limited quality of hospital routine data in Mali. The case definitions used by the health system are more oriented to allow the provision of prompt care, and the specificity of case definition is of less importance. Finally, the case-control study initially recruited participants only up to 5 years of age. This may have affected the distribution of severe malaria phenotypes observed.

Given the importance of cerebral malaria's direct and indirect effects on populations living in malaria-endemic regions, it is critical to identify measures that reduce its occurrence. Despite the limits of our approach, we observed an increased frequency of SMA and fewer cases of CM in the study settings as well as age shifts in severe malaria burden. These findings should be confirmed by studies done in other areas with seasonal malaria transmission and widespread malaria control measures, including SMC implemented at scale.

## Conclusion

Increased implementation of malaria control strategies was associated with a change in the clinical expression of severe malaria, including more severe malarial anemia and fewer cerebral malaria cases than expected as well as age shifts in severe malaria burden. Studies in different settings with various patterns of malaria transmission are needed to confirm the changes reported here and to characterize the clinical epidemiology of severe malaria in the context of scaling up of control strategies.

## Data availability statement

The original contributions presented in the study are included in the article/Supplementary material, further inquiries can be directed to the corresponding author.

## Ethics statement

The studies involving human participants were reviewed and approved by the Faculty of Medicine, Pharmacy, and Odonto-Stomatology (FMPOS) Ethics Committee. This Institutional Review Board is affiliated with the Faculty of Medicine of the University of Science, Techniques, and Technologies of Bamako (USTTB) and has a Federal wide Assurance with United States HHS for the Protection of Human subject FWA00001769. Written informed consent to participate in this study was provided by the participant's parents/legal guardian.

## Author contributions

MATh, DC, OD, and MATr conceived and designed the study. DC, AK, BK, BG, BT, MS, FM, KT, AD, ATr, and ATh collected the data in Bandiagara, Bamako, and Sikasso. MSS analyzed the data. MATh, DC, and MATr interpreted the data and results. MATh wrote the first draft of the manuscript. All authors reviewed and approved the submitted manuscript.

## Funding

This work was supported by the National Institute of Allergy and Infectious Diseases of the National Institutes of Health under Award Number R01AI099628 and grants R01HL130750 and R01HL146377 from the National Heart, Lung, and Blood Institute. This research program is part of the EDCTP2 programme supported by the European Union.

## Conflict of interest

The authors declare that the research was conducted in the absence of any commercial or financial relationships that could be construed as a potential conflict of interest.

## Publisher's note

All claims expressed in this article are solely those of the authors and do not necessarily represent those of their affiliated organizations, or those of the publisher, the editors and the reviewers. Any product that may be evaluated in this article, or claim that may be made by its manufacturer, is not guaranteed or endorsed by the publisher.

## Author disclaimer

The content is solely the responsibility of the authors and does not necessarily represent the official views of the National Institutes of Health.
