# Supplementary material for: Shifts in the clinical epidemiology of severe malaria after scaling up control strategies in Mali
Source: Front Neurol. 2022 Nov 29;13:988960. doi: 10.3389/fneur.2022.988960 (PMC9744791; doi:10.3389/fneur.2022.988960)
Supplement: Supplementary file 1 [file Table_1.pdf]

Supplementary Table 1: Distribution of severe malaria cases by Blantyre Coma Score (BCS) on day 0 (day of diagnosis) and after recovery.

Blantyre score	Day 0	After recovery
1	20	0
2	27	0
3	10	0
4	3	1
5	37	80
Total	97	81

The Blantyre Coma Score (BSC) was not collected for 16 children after recovery, including for the nine cases that died.
