# Supplementary material for: Shifts in the clinical epidemiology of severe malaria after scaling up control strategies in Mali
Source: Front Neurol. 2022 Nov 29;13:988960. doi: 10.3389/fneur.2022.988960 (PMC9744791; doi:10.3389/fneur.2022.988960)
Supplement: Supplementary file 2 [file Table_2.pdf]

Supplementary Table 2: Distribution of cases with severe malaria anemia by parasite density in the case-control study.

Clinical Phenotype	Parasite density				Total
	<10000	10001-50000	50001-99999	≥ 100000	
	n	n	n	n	
SMA only	18	14	8	8	48
SMA+CM	9	1	1	3	14
SMA+CM+Hypoglycemia	2	1	0	1	4
Total	30	16	9	12	67

Most severe malaria anemia cases are associated with *P. falciparum* parasite density below 10,000 parasites per µL.
