# Supplementary material for: Shifts in the clinical epidemiology of severe malaria after scaling up control strategies in Mali
Source: Front Neurol. 2022 Nov 29;13:988960. doi: 10.3389/fneur.2022.988960 (PMC9744791; doi:10.3389/fneur.2022.988960)
Supplement: Supplementary file 3 [file Table_3.pdf]

Supplementary Table 3: Distribution of cases with other mixed severe malaria clinical phenotypes in the National Hospital of Mali records survey.

Clinical Phenotypes	Pre SMC [2013-2015] (N=86)	Post SMC [2016-2019] (N=102)
	n	n
CM + Dehydration	11	3
Hypoglycemia	17	4
CM + Hypoglycemia	13	16
SMA + Hypoglycemia	15	14
CM + SMA + Hypoglycemia	12	3
Dehydration	6	11
SMA + Dehydration	3	2
CM + Hyperparasitemia	1	16
SMA + Hyperparasitemia	3	9
DIVC	2	2
Hyperparasitemia	2	4
CM + Respiratory distress	0	4
SMA + Respiratory distress	0	2
Mixed syndromes	1	12
Total	86	102

DIVC= Disseminated Intravascular Coagulation

Mixed syndromes are different combinations including two, three or four of the following: CM, SMA, hyperparasitemia, hypoglycemia, Respiratory distress, DIVC, and Dehydration.
