# Supplementary figures and images for: Shifts in the clinical epidemiology of severe malaria after scaling up control strategies in Mali
Source: Front Neurol. 2022 Nov 29;13:988960. doi: 10.3389/fneur.2022.988960 (PMC9744791; doi:10.3389/fneur.2022.988960)

# CARTE DU MALI

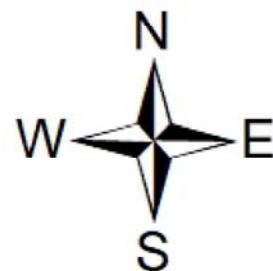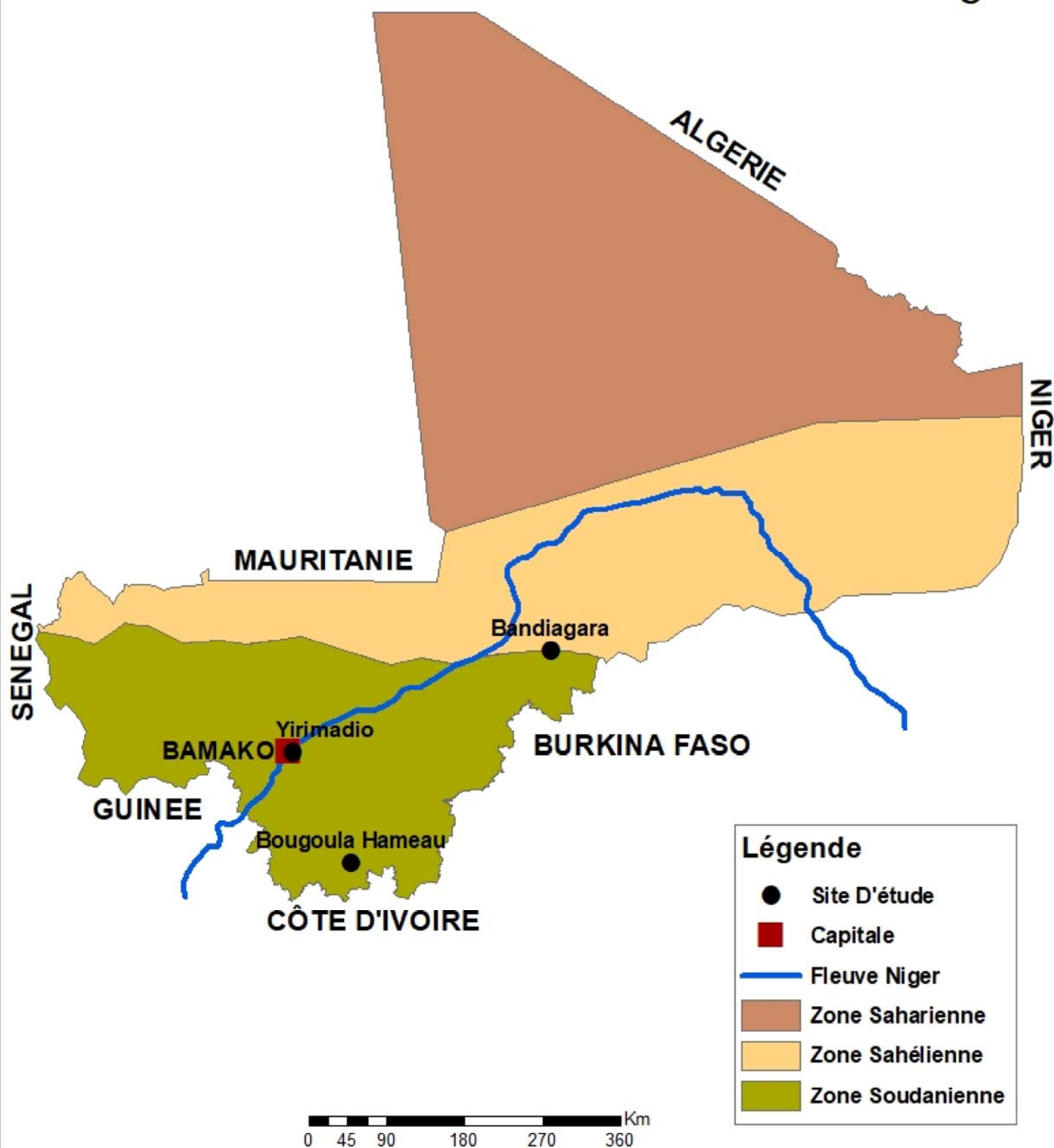

Supplement: Supplementary file 4 [file Image_1.pdf]

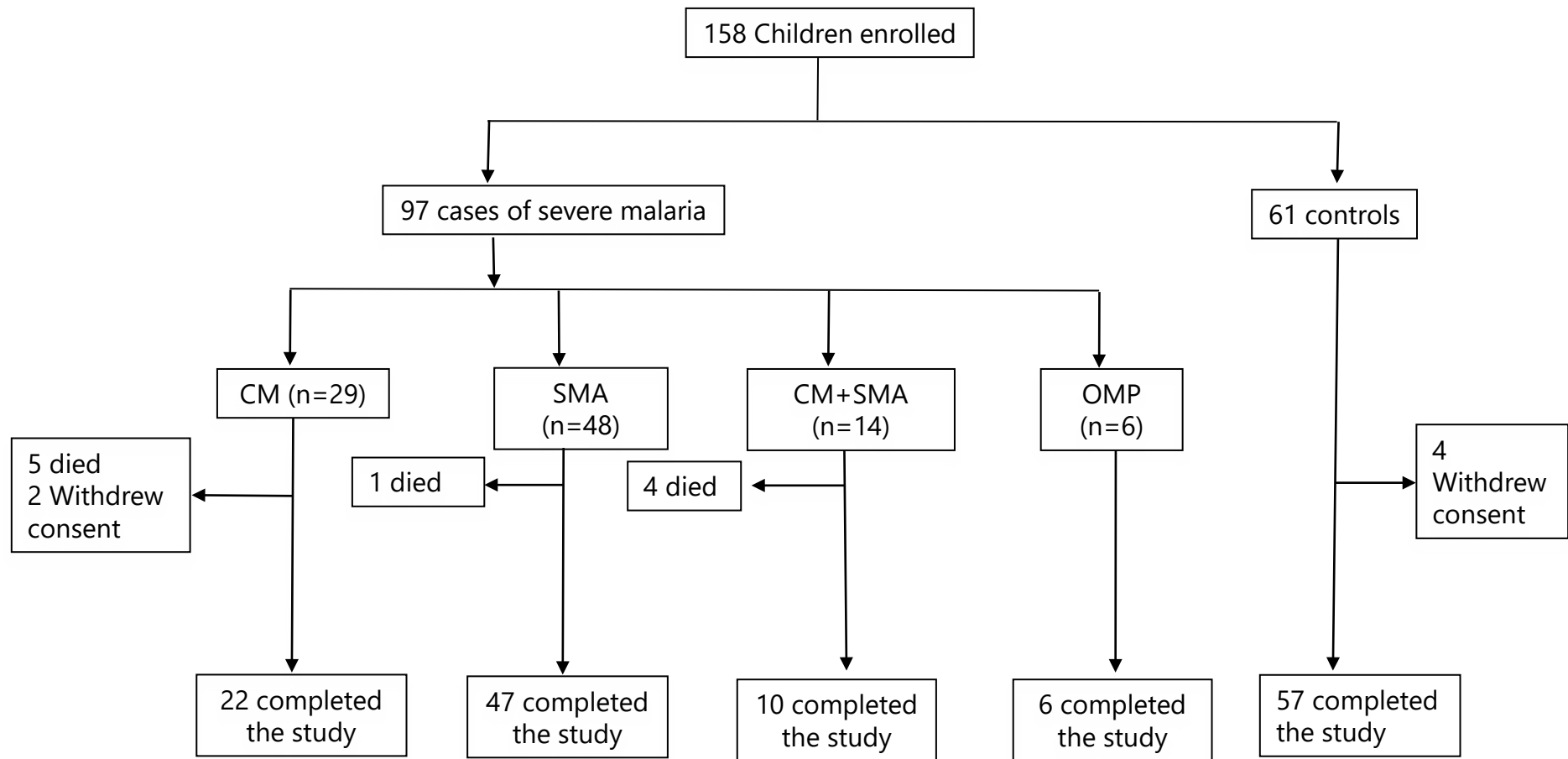

**Supplementary Figure 2: Consort diagram of the case-control study**

Supplement: Supplementary file 5 [file Image_2.pdf]
